# HLA-*DR3* ~ *DQ2* associates with sensory neuropathy in paraneoplastic neurological syndromes with Hu antibodies

**DOI:** 10.1007/s00415-024-12534-7

**Published:** 2024-07-11

**Authors:** Sergio Muñiz-Castrillo, Macarena Villagrán-García, Vicente Peris Sempere, Antonio Farina, Anne-Laurie Pinto, Géraldine Picard, Véronique Rogemond, Jérôme Honnorat, Emmanuel Mignot

**Affiliations:** 1 Stanford Center for Sleep Sciences and Medicine, Stanford University, 3165 Porter Drive, Palo Alto, CA 94304 USA; 2 French Reference Center for Paraneoplastic Neurological Syndromes and Autoimmune Encephalitis, Hospices Civils de Lyon, Lyon, France; 3 MeLiS, UCBL-CNRS UMR 5284, INSERM U1314, Université Claude Bernard Lyon 1, Lyon, France

**Keywords:** Paraneoplastic neurological syndromes, Hu antibody, Small-cell lung cancer, HLA

## Abstract

**Objectives:**

To investigate the association between human leukocyte antigen (HLA) and paraneoplastic neurological syndromes (PNS) with Hu antibodies, and potential specificities according to clinical presentation and cancer status.

**Methods:**

HLA genotypes at four-digit resolution were imputed from available genome-wide association data. Allele carrier frequencies were compared between patients (whole cohort, *n* = 100, and according to clinical presentation and cancer status) and matched healthy controls (*n* = 508) using logistic regression controlled by the three main principal components.

**Results:**

The clinical presentation of 100 anti-Hu patients involved the central nervous system (28, 28%), the peripheral nervous system (36, 36%) or both combined (36, 36%). Cancer diagnosis was certain in 75 (75%). HLA association analyses revealed that anti-Hu PNS patients were more frequently carriers of DQA1*05:01 (39% vs. 19%, OR = 2.8 [1.74–4.49]), DQB1*02:01 (39% vs. 18%, OR = 2.88 [1.79–4.64]) and DRB1*03:01 (41% vs. 19%, OR = 2.92 [1.80–4.73]) than healthy controls. Remarkably, such *DR3* ~ *DQ2* association was absent in patients with pure central involvement, but more specific to those manifesting with peripheral involvement: DQA1*05:01 (OR = 3.12 [1.48–6.60]), DQB1*02:01 (OR = 3.35 [1.57–7.15]) and DRB1*03:01 (OR = 3.62 [1.64–7.97]); being even stronger in cases with sensory neuropathy, DQA1*05:01 (OR = 4.41 [1.89–10.33]), DQB1*02:01 (OR = 4.85 [2.04–11.53]) and DRB1*03:01 (OR = 5.79 [2.28–14.74]). Similarly, *DR3* ~ *DQ2* association was only observed in patients with cancer.

**Discussion:**

Patients with anti-Hu PNS show different HLA profiles according to clinical presentation and, probably, cancer status, suggesting pathophysiological differences.

**Supplementary Information:**

The online version contains supplementary material available at 10.1007/s00415-024-12534-7.

## Introduction

Paraneoplastic neurological syndromes (PNS) with Hu antibodies are strongly associated with small-cell lung cancer (SCLC), but show a heterogenous clinical presentation, ranging from isolated peripheral (e.g., sensory neuropathy) or central (e.g., limbic encephalitis) nervous system disorders, to, very often, multifocal involvement known as encephalomyelitis [1, 2]. The reason underlying this clinical diversity, and whether it reflects different pathophysiological pathways, is yet unknown. Moreover, why immune tolerance breaks down in SCLC and develops anti-Hu PNS is not understood. Indeed, SCLC constantly expresses Hu antigens, but only ~ 15% of patients harbor Hu antibodies and even fewer manifest with PNS [3]. Recent studies in other PNS have described particular genetic features of the associated tumors, but, conversely, no mutations in Hu genes have been identified in SCLC to date [4]. Investigation into whether the genetic characteristics of the patients themselves could play a role in the pathogenesis of anti-Hu PNS has so far been limited to a few small case series focusing on the human leukocyte antigen (HLA) [5, 6]. Herein, we conducted an HLA association analysis in a large cohort of anti-Hu PNS, exploring potential differences according to clinical presentation and cancer status.

## Methods

### Patients and clinical classification

From a total of 466 patients with anti-Hu PNS from the French Reference Center for PNS and Autoimmune Encephalitis (1990–2022), 100 (21%) had available DNA and were included in the study. The clinical picture was characterized according to: (1) general nervous system involvement (i.e., central nervous system, peripheral nervous system or combined) and (2) PNS phenotype (i.e., limbic encephalitis, brainstem/cerebellar, sensory neuropathy that included sensory neuronopathy and other less specific patterns after exclusion of alternative causes, motor neuropathy, Lambert–Eaton myasthenic syndrome or multifocal whenever two or more phenotypes existed). We coded patients as having "sensory neuropathy" following the approach of previous researchers who used this term for patients with a predominant large fiber sensory neuropathy, even with mild motor symptoms [1]. This decision stems from the difficulty of obtaining consistent electrophysiology evaluations in a retrospective cohort and evidence showing that most anti-Hu PNS patients with clinically pure sensory neuronopathy also had motor abnormalities [7]. The classification of cancer status was based on three categories: whether an established cancer diagnosis was obtained, or alternatively, if the length of follow-up exceeded or was less than 2 years.

### HLA analysis

Patients and 508 healthy controls provided by the Stanford Center for Sleep Sciences and Medicine were genotyped using the Affymetrix PMRA array. Genotypes were processed using PLINK (version 2.0) as previously reported [8], and a principal component analysis (PCA), Euclidean distance-based measure was used to match patients to the closest controls (ratio 1:10, Supplementary Figure). HLA imputation was performed using HLA Genotype Imputation with Attribute Bagging [9]. HLA genotypes with an imputation probability lower than 0.3 were excluded. Allele carrier frequencies were first compared between patients (entire cohort and according to clinical presentation and cancer status) and PCA-matched controls using logistic regression controlled by the three main PCs. Secondly, a logistic regression controlled by the three main PCs and the significant alleles found in the first analysis was performed. Additional analyses comprised a zygosity analysis for the effect of *DR3* ~ *DQ2* haplotype dosage and a logistic regression in non-*DR3* ~ *DQ2* carriers. Multiple comparisons were corrected by Bonferroni’s method, and corrected *p* values < 0.05 were considered statistically significant. HLA analyses were performed using R Studio (version 2023.12.1 + 402).

### Ethics approval

The study was approved by the Institutional Review Boards of Stanford University (IGNITE, IRB-65073) and Université Claude Bernard Lyon 1 and Hospices Civils de Lyon (ICARE-II, NCT04823728). Written informed consent was obtained from all participants for the storage and use of biological samples and clinical information for research purposes. The study was performed in accordance with the ethical standards framed by the Declaration of Helsinki and its later amendments.

## Results

### Demographic and clinical features

**Fig. 1:**
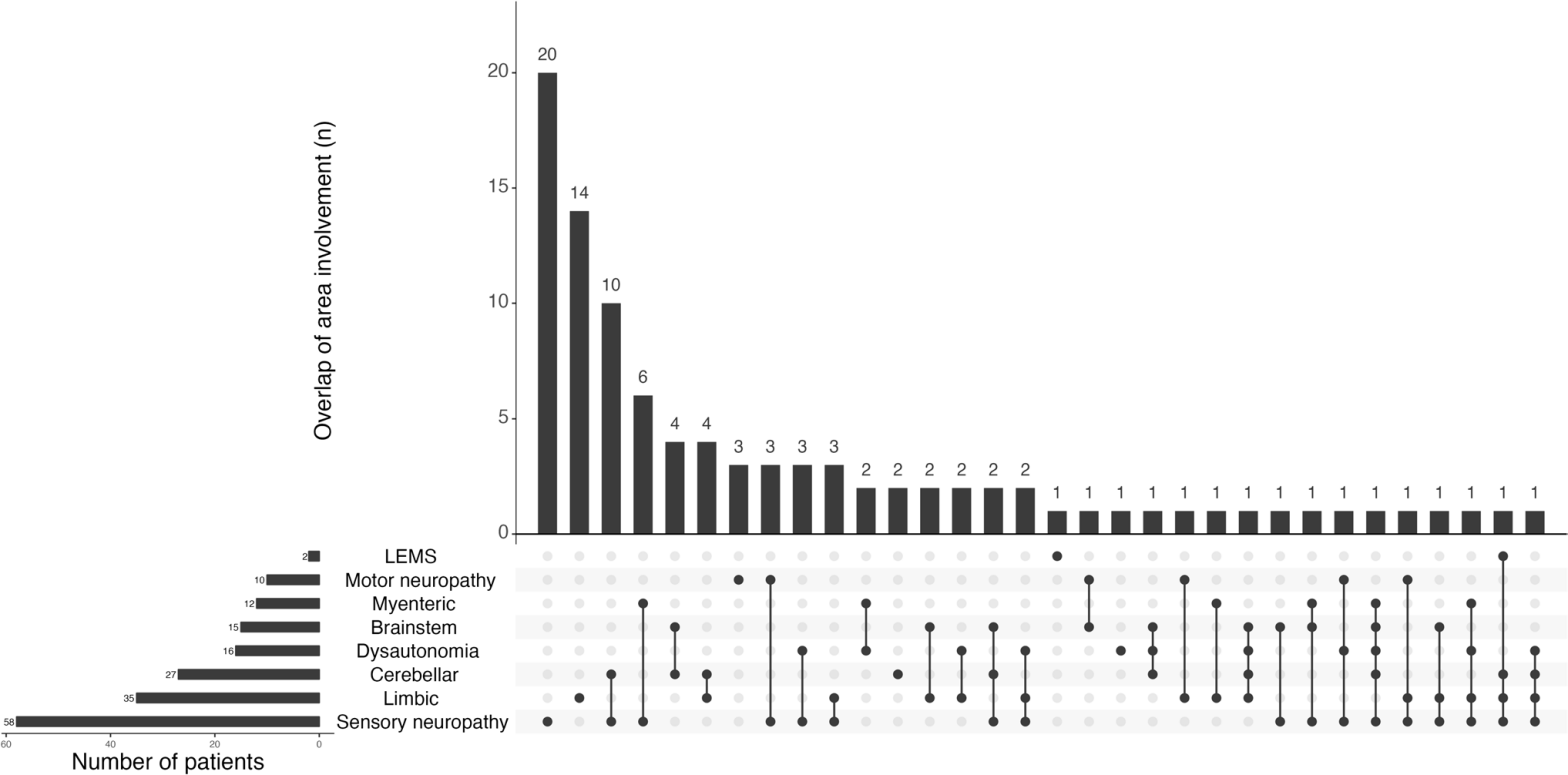
Upset plot of the isolated and combined phenotypes in 100 patients with anti-Hu PNS. Each row represents a clinical involvement, and the total number of patients with each of them are represented in the left horizontal bar chart. The columns represent the number of patients with isolated or combined clinical pictures. The cells are filled in black whenever a phenotype is present, and the dots are connected by a line when several phenotypes overlap

The main demographic and clinical features of the 100 patients with anti-Hu PNS are summarized in the Table 1. Notably, clinical presentation involved the central (28, 28%), peripheral nervous system (36, 36%) or both combined (36, 36%); the diversity and overlap of PNS phenotype are depicted in Fig. 1. As expected, among the 75 (75%) patients with a diagnosis of cancer, 62 (83%) were SCLC.

**Table 1:** Main demographic and clinical features of the cohort

Clinical features	*n* = 100
Age, median (IQR)	64 (57, 70)
Sex, *n* (%)	
Female	49 (49)
Male	51 (51)
Cancer status, *n* (%)	
Cancer diagnosis	75 (75)
No cancer (< 2 year follow-up)	13 (13)
No cancer (≥ 2 year follow-up)	12 (12)
Cancer type, *n* (%)	
SCLC	62 (83)
NSCLC	6 (8)
Others^a^	7 (9)
Symptom chronology, *n* (%)	
Symptoms preceding cancer/no cancer	87 (87)
Symptoms after cancer	9 (9)
Post-ICI	4 (4)
Type of symptom onset, *n* (%)	98 (98)
Acute	8 (8)
Subacute	69 (70)
Chronic	21 (21)
Nervous system involvement, *n* (%)	
Central	28 (28)
Combined	36 (36)
Peripheral	36 (36)
Phenotype, *n* (%)	
Limbic	13 (13)
Brainstem/cerebellar^b^	6 (6)
Sensory neuropathy	28 (28)
Motor neuropathy	3 (3)
LEMS	1 (1)
Multifocal^c^	49 (49)
Coexistent neural antibody^d^, *n* (%)	18 (18)

^a^Breast cancer: *n* = 2, gastrointestinal cancer *n* = 2, prostate cancer *n* = 1, bladder cancer *n* = 1, undifferentiated small cell cancer of unknown origin *n* = 1

^b^Isolated cerebellitis: *n* = 2, brainstem encephalitis and cerebellar ataxia n = 4

^c^Limbic involvement: *n* = 22, brainstem *n* = 11, cerebellar *n* = 21, sensory neuropathy *n* = 32, motor neuropathy *n* = 7, myenteric *n* = 4, dysautonomia *n* = 9, LEMS *n* = 1

^d^SOX1: *n* = 7, CV2 *n* = 5, ZIC4 *n* = 4, amphyphisin *n* = 2, GAD65 n = 2, AMPAR *n* = 1 (several autoantibodies could coexist in the same patient)

### HLA analysis in the whole cohort

**Fig. 2:**
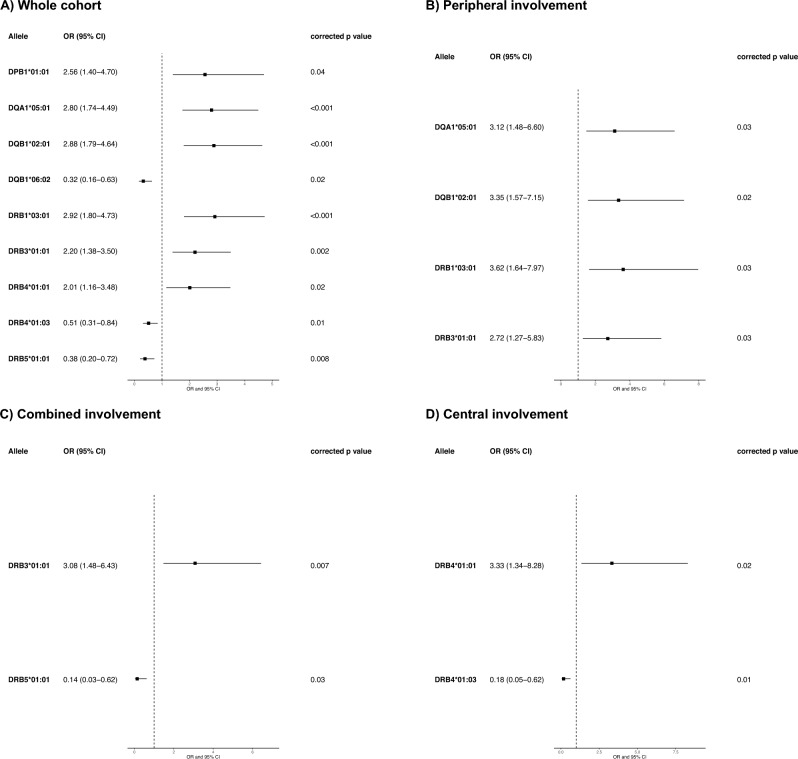
HLA association study in the whole anti-Hu PNS cohort and according to clinical involvement. Forest plot depicting the significant HLA allele associations after performing logistic regression controlling for the three main principal components on: **A** the whole cohort of patients with PNS and Hu antibodies (*n* = 100), **B** patients with peripheral involvement (*n* = 36), patients with combined involvement (*n* = 36) and those with exclusively central involvement (*n* = 28)

Patients with anti-Hu PNS were significantly more frequently carriers of DPB1*01:01 (OR = 2.56 [1.40–4-70], corrected *p* value = 0.03), DQA1*05:01 (OR = 2.80 [1.74–4.49], corrected *p* value = 0.00019), DQB1*02:01 (OR = 2.88 [1.79–4.64], corrected *p* value = 0.00015, DRB1*03:01 (OR = 2.92 [1.80–4.73], corrected *p* value = 0.00031), DRB3*01:01 (OR = 2.20 [1.38–3.50], corrected *p* value = 0.02) and DRB4*01:01 (OR = 2.01 [1.16–3.48], corrected p value = 0.00031), in comparison to healthy controls (Fig. 2A, Supplementary Table 1). Noteworthy, DPB1*01:01 ~ DQA1*05:01 ~ DQB1*02:01 ~ DRB1*03:01 ~ DRB3*01:01 constitutes a common conserved haplotype (*DR3* ~ *DQ2*) [10]. Additionally, protective effects were identified for DQB1*06:02, DRB4*01:03 and DRB5*01:01 (Fig. 2A, Supplementary Table 1).

Logistic regression, including as covariates the significant alleles, showed that the DPB1*01:01 effect disappeared when controlling for the rest of the alleles of the *DR3* ~ *DQ2* haplotype, but was unable to determine whether a DR or DQ effect is responsible for the *DR3* ~ *DQ2* predisposition (Supplementary Table 2). In addition, the predisposing effect of DRB4*01:01, an allele carried by a subset of DRB1*07:01 ~ DQB1*02:02 haplotypes, remained after controlling for the alleles of the *DR3* ~ *DQ2* haplotype (Supplementary Table 3). Furthermore, no zygosity effect was observed for the *DR3* ~ *DQ2* haplotype (Supplementary Table 4). Logistic regression in non-*DR3* ~ *DQ2* carriers also showed a predisposing effect for DRB4*01:01, along with DRB3*02:02; DRB4*01:03 was still identified as a protective allele in this subgroup (Supplementary Table 5).

### HLA analysis according to clinical presentation

**Fig. 3:**
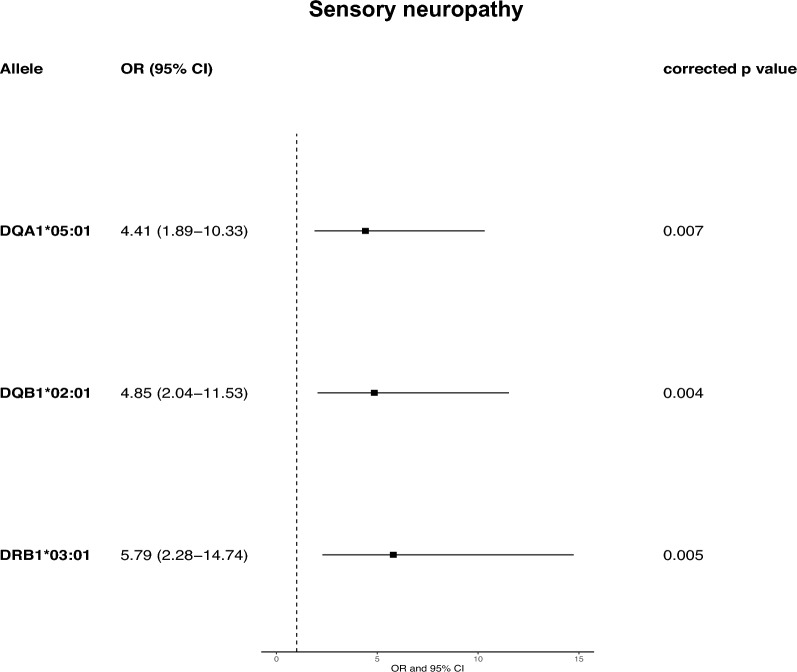
HLA-*DR3* ~ *DQ2* associates with sensory neuropathy in anti-Hu PNS. Forest plot depicting the significant HLA allele associations after performing logistic regression controlling for the three main principal components in patients with sensory neuropathy (*n* = 28)

The association with DQA1*05:01 ~ DQB1*02:01 ~ DRB1*03:01 ~ DRB3*01:01 was also observed when patients with anti-Hu PNS and peripheral involvement were analyzed separately (Fig. 2B, Supplementary Table 6). Conversely, among patients with combined involvement, only DRB3*01:01 remained statistically significant (Fig. 2C, Supplementary Table 7), whereas the association with the *DR3* ~ *DQ2* haplotype was absent in patients with central involvement (Fig. 2D, Supplementary Table 8). Since patients with peripheral involvement comprised cases with either sensory neuropathy, motor neuropathy or Lambert–Eaton myasthenic syndrome, we then analyzed only those presenting with sensory neuropathy, confirming an association with DQA1*05:01 ~ DQB1*02:01 ~ DRB1*03:01 (Fig. 3, Supplementary Table 9).

### HLA analysis according to *cancer* status

Patients with anti-Hu PNS and a diagnosed cancer showed similar HLA associations than those observed when the entire cohort was analyzed, with predisposing effects for A*34:02, DPB1*01:01, DQA1*05:01, DQB1*02:01, DQB1*02:02, DRB1*03:01, DRB3*01:01 and DRB4*01:01, and a protective effect for DRB4*01:03 (Supplementary Table 10). The results were almost identical when only patients with SCLC were investigated, with a risk effect for A*34:02, DQA1*02:01, DQA1*05:01, DQB1*02:01, DQB1*02:02, DRB1*03:01, DRB3*01:01 and DRB4*01:01 (Supplementary Table 11). Logistic regression performed on the 12 patients with no cancer after > 2 years of follow-up did not identify any significant HLA association (data not shown), but it is noteworthy that only 2 (17%) were *DR3* ~ *DQ2* carriers.

## Discussion

Herein, we confirm an association between anti-Hu PNS and the HLA haplotype *DR3 ~ DQ2* previously reported in 53 patients [6], which was carried by nearly 40% of the patients of the present cohort of 100 individuals. This association is not related to the underlying cancer as SCLC does not show any HLA association [11]. Most interestingly, we also found that this association is more specific to patients with peripheral involvement, and, particularly, those manifesting with sensory neuropathy. Conversely, cases with exclusive central involvement lacked the *DR3 ~ DQ2* association.

Along with the *DR3 ~ DQ2* association, we also found a secondary association with DQA1*02:01 ~ DQB1*02:02 ~ DRB4*01:01, which was more evident when only patients with cancer or SCLC were analyzed. Remarkably, DRB4*01:01 was the single predisposing allele detected in patients with central involvement. Although the epitope reactivity of Hu antibodies has not been observed to vary according to the clinical presentation [12, 13], the different immunogenetic profiles exhibited herein by the clinical phenotypes likely reflect that the underlying pathophysiological mechanisms might be, at least partially, distinct. Similarly, patients without an identified cancer lacked the *DR3 ~ DQ2* association, which could also suggest pathogenic differences compared to those with SCLC; however, it is noteworthy that the number of non-paraneoplastic cases analyzed was considerably small. Such clinical and oncological correlations with HLA have already been described in other PNS and related autoimmune encephalitis, like Lambert–Eaton myasthenic syndrome [14] or syndromes with contactin-associated protein-like 2 (CASPR2) antibodies [15]; nevertheless, in the aforementioned diseases, the non-paraneoplastic subtype is the one associated with HLA.

The haplotype *DR3 ~ DQ2* (and the extended ancestral haplotype 8.1, which also includes HLA class I alleles HLA-A*01:01, B*08:01, C*07:01 and class II DRB3*01:01) has been associated with many autoimmune diseases in populations of European descent, such as type 1 diabetes mellitus, celiac disease and myasthenia gravis [16]. The lack of antigen specificity suggests that other mechanisms different from altered peptide presentation could also be involved in the predisposition to autoimmunity conferred by the haplotype 8.1, and, accordingly, several immune dysfunctions have been reported in its carriers [16]. Furthermore, the haplotype *DR3 ~ DQ2* has been related to a few autoimmune encephalitis and PNS, principally to neurological syndromes with antibodies against glutamic acid decarboxylase 65 (GAD65) and limbic encephalitis with adenylate kinase 5 (AK5) antibodies [17, 18]. Interestingly, anti-Hu PNS, anti-GAD65 neurological syndromes and anti-AK5 limbic encephalitis seem to share a mostly CD8^+^ T cell-mediated pathogenesis [18–20]. It is therefore striking that no HLA class I association has been found for these diseases, although this could be due to the small sample size of the cohorts analyzed. In addition, CD4^+^ T cells might also be relevant to the immunopathogenesis of such disorders, as suggested by some neuropathological studies that showed important CD4^+^ T cell infiltrates accompanying CD8^+^ T cells [18, 21]. Remarkably, peripheral blood mononuclear cells (PBMCs) from patients with anti-Hu PNS stimulated with recombinant HuD protein (the main Hu antigen) exhibited an intense proliferation of CD4^+^ T cells, but not of CD8^+^ T cells [22]. These findings suggest that the activation of auto-reactive anti-Hu CD4^+^ T cells might be a necessary and early step in the pathogenesis of anti-Hu PNS, whereas the contribution of cytotoxic CD8^+^ T cells might occur later in the disease [22].

The main limitation of our study is the relatively small sample size, particularly when considering subgroups according to clinical involvement and phenotype. This point might also have hindered the identification of HLA associations in the non-paraneoplastic subset of patients.

In conclusion, we confirm an association with HLA-*DR3 ~ DQ2* and show that it preferentially associates with paraneoplastic sensory neuropathy and Hu antibodies, suggesting pathophysiological heterogeneity. Larger studies are warranted to better define the HLA association in anti-Hu PNS, as well as to explore the role of other non-HLA genes.

## Supplementary Information

Below is the link to the electronic supplementary material.

Supplementary file1 (XLSX 39 KB)

Supplementary Figure. Scatter plot showing the distribution of the two first principal components (PC1 and PC2) obtained from GWAS data in patients and controls. Each patient was matched with 10 ethnically similar controls for the HLA analysis. The large cluster of individuals (bottom left) corresponds to White Europeans, while those in the top right are of African descent, and those in the bottom right are of East Asian descent. Individuals situated between these three clusters represent ethnic admixture

## Data Availability

Anonymized data not published within this article will be made available by request from any qualified investigator.

## References

[1] Graus F (2001) Anti-Hu-associated paraneoplastic encephalomyelitis: analysis of 200 patients. Brain 124:1138–1148. 10.1093/brain/124.6.1138 11353730 10.1093/brain/124.6.1138

[2] Villagrán-García M, Farina A, Muñiz-Castrillo S et al (2023) Revisiting anti-Hu paraneoplastic autoimmunity: phenotypic characterization and cancer diagnosis. Brain Commun 5:247. 10.1093/braincomms/fcad247 10.1093/braincomms/fcad247 PMC10546956 37794924

[3] Dalmau J, Furneaux HM, Gralla RJ et al (1990) Detection of the anti-Hu antibody in the serum of patients with small cell lung cancer–a quantitative western blot analysis. Ann Neurol 27:544–552. 10.1002/ana.410270515 2163235 10.1002/ana.410270515

[4] Vogrig A, Pegat A, Villagrán-García M et al (2023) Different genetic signatures of small-cell lung cancer characterize anti-GABAB R and anti-Hu paraneoplastic neurological syndromes. Ann Neurol 94:1102–1115. 10.1002/ana.26784 37638563 10.1002/ana.26784

[5] Uchuya M, Fleury A, Graus F et al (1998) Lack of association between human leukocyte antigens and the anti-Hu syndrome in patients with small-cell lung cancer. Neurology 50:565–566. 10.1212/WNL.50.2.565 9484403 10.1212/WNL.50.2.565

[6] de Graaf MT, de Beukelaar JWK, Haasnoot GW et al (2010) HLA-DQ2+ individuals are susceptible to Hu-Ab associated paraneoplastic neurological syndromes. J Neuroimmunol 226:147–149. 10.1016/j.jneuroim.2010.05.035 20547426 10.1016/j.jneuroim.2010.05.035

[7] Camdessanché J-P, Antoine J-C, Honnorat J et al (2002) Paraneoplastic peripheral neuropathy associated with anti-Hu antibodies. A clinical and electrophysiological study of 20 patients. Brain 125:166–175. 10.1093/brain/awf006 11834602 10.1093/brain/awf006

[8] Peris Sempere V, Muñiz-Castrillo S, Ambati A et al (2022) Human leukocyte antigen association study reveals DRB1*04:02 effects additional to DRB1*07:01 in anti-LGI1 encephalitis. Neurol Neuroimmunol Neuroinflamm 9:e1140. 10.1212/NXI.0000000000001140 35115410 10.1212/NXI.0000000000001140 PMC8815287

[9] Zheng X, Shen J, Cox C et al (2014) HIBAG—HLA genotype imputation with attribute bagging. Pharmacogenomics J 14:192–200. 10.1038/tpj.2013.18 23712092 10.1038/tpj.2013.18 PMC3772955

[10] Osoegawa K, Mallempati KC, Gangavarapu S et al (2019) HLA alleles and haplotypes observed in 263 US families. Hum Immunol 80:644–660. 10.1016/j.humimm.2019.05.018 31256909 10.1016/j.humimm.2019.05.018 PMC6773484

[11] Enjo-Barreiro JR, Ruano-Ravina A, Pérez-Ríos M et al (2024) Genome wide association studies in small-cell lung cancer. A systematic review. Clin Lung Cancer 25:9–17. 10.1016/j.cllc.2023.10.002 37940411 10.1016/j.cllc.2023.10.002

[12] Graus YF, Verschuuren JJ, Degenhardt A et al (1998) Selection of recombinant anti-HuD Fab fragments from a phage display antibody library of a lung cancer patient with paraneoplastic encephalomyelitis. J Neuroimmunol 82:200–209. 10.1016/s0165-5728(97)00199-9 9585817 10.1016/s0165-5728(97)00199-9

[13] Sodeyama N, Ishida K, Jaeckle KA et al (1999) Pattern of epitopic reactivity of the anti-Hu antibody on HuD with and without paraneoplastic syndrome. J Neurol Neurosurg Psychiatry 66:97–99. 10.1136/jnnp.66.1.97 9886463 10.1136/jnnp.66.1.97 PMC1736149

[14] Wirtz PW, Willcox N, van der Slik AR et al (2005) HLA and smoking in prediction and prognosis of small cell lung cancer in autoimmune Lambert-Eaton myasthenic syndrome. J Neuroimmunol 159:230–237. 10.1016/j.jneuroim.2004.10.018 15652424 10.1016/j.jneuroim.2004.10.018

[15] Muñiz-Castrillo S, Joubert B, Elsensohn M-H et al (2020) Anti-CASPR2 clinical phenotypes correlate with HLA and immunological features. J Neurol Neurosurg Psychiatry 91:1076–1084. 10.1136/jnnp-2020-323226 32651251 10.1136/jnnp-2020-323226

[16] Gambino CM, Aiello A, Accardi G et al (2018) Autoimmune diseases and 8.1 ancestral haplotype: an update. HLA 92:137–143. 10.1111/tan.13305 29877054 10.1111/tan.13305

[17] Muñiz-Castrillo S, Ambati A, Dubois V et al (2020) Primary DQ effect in the association between HLA and neurological syndromes with anti-GAD65 antibodies. J Neurol 267:1906–1911. 10.1007/s00415-020-09782-8 32152690 10.1007/s00415-020-09782-8

[18] Muñiz-Castrillo S, Hedou JJ, Ambati A et al (2021) Distinctive clinical presentation and pathogenic specificities of anti-AK5 encephalitis. Brain. 10.1093/brain/awab153 33843981 10.1093/brain/awab153 PMC8557339

[19] Bien CG, Vincent A, Barnett MH et al (2012) Immunopathology of autoantibody-associated encephalitides: clues for pathogenesis. Brain 135:1622–1638. 10.1093/brain/aws082 22539258 10.1093/brain/aws082

[20] Roberts WK, Deluca IJ, Thomas A et al (2009) Patients with lung cancer and paraneoplastic Hu syndrome harbor HuD-specific type 2 CD8+ T cells. J Clin Investig. 10.1172/JCI36131 19509467 10.1172/JCI36131 PMC2701858

[21] Jean WC, Dalmau J, Ho A, Posner JB (1994) Analysis of the IgG subclass distribution and inflammatory infiltrates in patients with anti-Hu-associated paraneoplastic encephalomyelitis. Neurology 44:140–147. 10.1212/wnl.44.1.140 8290049 10.1212/wnl.44.1.140

[22] Benyahia B, Liblau R, Merle-Béral H et al (1999) Cell-mediated autoimmunity in paraneoplastic neurological syndromes with anti-Hu antibodies. Ann Neurol 45:162–167. 10.1002/1531-8249(199902)45:2%3c162::aid-ana5%3e3.0.co;2-r 9989617 10.1002/1531-8249(199902)45:2<162::aid-ana5>3.0.co;2-r

