# Supplementary figures and images for: HLA-DR3 ~ DQ2 associates with sensory neuropathy in paraneoplastic neurological syndromes with Hu antibodies
Source: J Neurol. 2024 Jul 11;271(9):6336–42. doi: 10.1007/s00415-024-12534-7 (PMC11377461; doi:10.1007/s00415-024-12534-7)

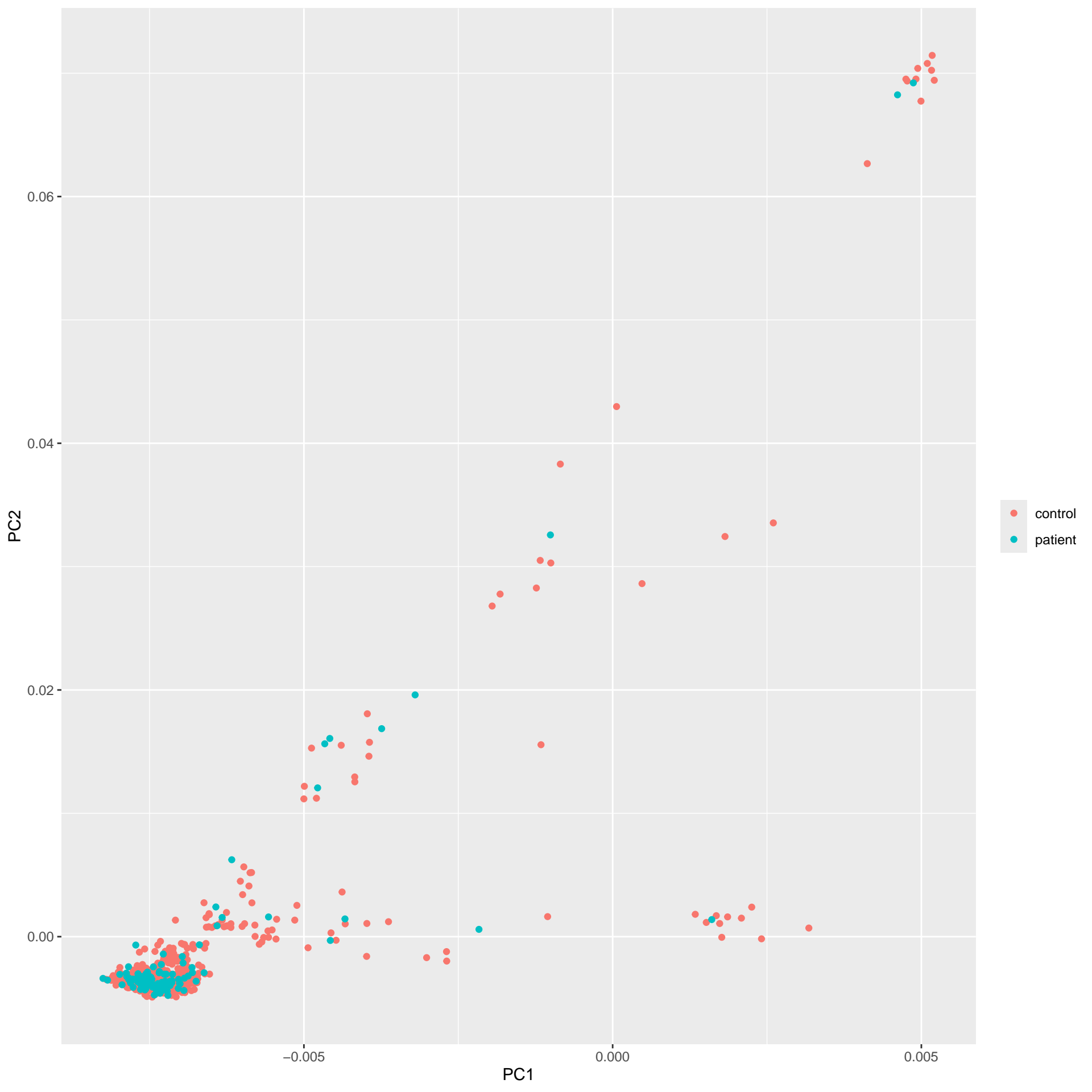

Supplement: Supplementary file 2 — Supplementary Figure. Scatter plot showing the distribution of the two first principal components (PC1 and PC2) obtained from GWAS data in patients and controls. Each patient was matched with 10 ethnically similar controls for the HLA analysis. The large cluster of individuals (bottom left) corresponds to White Europeans, while those in the top right are of African descent, and those in the bottom right are of East Asian descent. Individuals situated between these three clusters represent ethnic admixture [file 415_2024_12534_MOESM2_ESM.pdf]
